# Early Life Stress Induces Different Behaviors in Adolescence and Adulthood May Related With Abnormal Medial Prefrontal Cortex Excitation/Inhibition Balance

**DOI:** 10.3389/fnins.2021.720286

**Published:** 2022-01-04

**Authors:** Yiwen Chen, Yuanjia Zheng, Jinglan Yan, Chuanan Zhu, Xuan Zeng, Shaoyi Zheng, Wenwen Li, Lin Yao, Yucen Xia, Wei-wei Su, Yongjun Chen

**Affiliations:** ^1^South China Research Center for Acupuncture and Moxibustion, Medical College of Acu-Moxi and Rehabilitation, Guangzhou University of Chinese Medicine, Guangzhou, China; ^2^Research Institute of Acupuncture and Moxibustion, Shandong University of Traditional Chinese Medicine, Jinan, China; ^3^Department of Integrated Traditional Chinese and Western Medicine, Xiamen Xianyue Hospital, Xiamen, China; ^4^Guangdong Key Laboratory of Plant Resources, School of Life Sciences, Sun Yat-sen University, Guangzhou, China; ^5^Guangdong-Hong Kong-Macao Greater Bay Area Center for Brain Science and Brain-Inspired Intelligence, Guangzhou, China; ^6^Guangdong Province Key Laboratory of Psychiatric Disorders, Southern Medical University, Guangzhou, China

**Keywords:** early life stress, E/I balance, maternal separation, depression, glutamate release

## Abstract

Early life stress is thought to be a risk factor for emotional disorders, particularly depression and anxiety. Although the excitation/inhibition (E/I) imbalance has been implicated in neuropsychiatric disorders, whether early life stress affects the E/I balance in the medial prefrontal cortex at various developmental stages is unclear. In this study, rats exposed to maternal separation (MS) that exhibited a well-established early life stress paradigm were used to evaluate the E/I balance in adolescence (postnatal day P43–60) and adulthood (P82–100) by behavior tests, whole-cell recordings, and microdialysis coupled with high performance liquid chromatography-mass spectrometry (HPLC-MS) analysis. First, the behavioral tests revealed that MS induced both anxiety- and depressive-like behaviors in adolescent rats but only depressive-like behavior in adult rats. Second, MS increased the action potential frequency and E/I balance of synaptic transmission onto L5 pyramidal neurons in the prelimbic (PrL) brain region of adolescent rats while decreasing the action potential frequency and E/I balance in adult rats. Finally, MS increases extracellular glutamate levels and decreased the paired-pulse ratio of evoked excitatory postsynaptic currents (EPSCs) of pyramidal neurons in the PrL of adolescent rats. In contrast, MS decreased extracellular glutamate levels and increased the paired-pulse ratio of evoked EPSCs of pyramidal neurons in the PrL of adult rats. The present results reveal a key role of E/I balance in different MS-induced disorders may related to the altered probability of presynaptic glutamate release at different developmental stages.

## Introduction

Early life is a critical period for brain development, both in humans and rodents, during which neuronal plasticity, synaptic organization, and remodeling activities rapidly develop (Di Segni et al., 2018). Early life stress (ELS) is a type of trauma that is experienced early in life (infancy or adolescence), including domestic violence, physical or sexual abuse, negligence, parental mental illness, drug abuse, etc. Previous studies clearly showed that ELS is an important risk factor for several mental diseases, including depression, anxiety, posttraumatic stress disorder, and schizophrenia (Collishaw et al., 2007; Gibb et al., 2007; Coplan et al., 2014; Ishikawa et al., 2015). ELS has been shown to have a wide range of long-term effects on neuroendocrine signals, neuronal morphology and plasticity, and local brain volume and function (Chen and Baram, 2016; van Bodegom et al., 2017; Babicola et al., 2021). However, its impact on various developmental stages and its mechanism of action remains to be elucidated.

Excitatory and inhibitory synapses are the two most abundant types of synapses in the brain. Glutamatergic pyramidal neurons are excitatory, and their activity is coordinated by an intricate network of inhibitory GABAergic interneurons (Rossignol, 2011). The balance between excitatory and inhibitory synaptic transmission (E/I) is essential to ensure proper information processing and maintain a finely tuned balance in neural activity, which is vital for central physiological functions (Barden, 2004; Luscher et al., 2011). E/I imbalance contributes to numerous neuropsychiatric phenotypes, including anxiety, depression, and epilepsy (Yuen et al., 2012; Li et al., 2017; Czeh et al., 2018; Ghosal et al., 2020; Kim Y. et al., 2020); however, the effects of early stress on E/I balance at different developmental stages are still unclear.

The medial prefrontal cortex (mPFC), a crucial region that is implicated in the pathophysiology of emotional disorders (Liu et al., 2012; Zhang et al., 2017; Liang et al., 2018), is sensitive to stress exposure in early life (McKlveen et al., 2013, 2015). Several studies have shown that maternal separation (MS) induces an increase in 5-hydroxytryptamine transporter expression and decrease in brain-derived neurotrophic factor (BDNF) levels in rat mPFC (Park et al., 2012; Jiang et al., 2021; Tata et al., 2021). MS results in decreased neuron excitability and abnormal myelin formation in the mPFC (Liu et al., 2012). The mPFC has a high degree of connectivity with limbic brain areas such as the nucleus accumbens and amygdala, which play an important role in regulating emotional behavior (Calhoon and Tye, 2015; Sah, 2017; Muir et al., 2019). The mPFC includes several subregions such as the anterior cingulate and prelimbic (PrL) and infralimbic (IL) cortex, which have different functional connections with other brain regions and therefore play different roles in neuropsychiatric diseases (Heidbreder and Groenewegen, 2003). PrL is directly involved in cognitive processes, and damage to PrL leads to a delayed response to stress (Barreto et al., 2012). However, the specific effect of early stress on synaptic and neuronal activity in PrL at different developmental stages remains unknown.

We conducted a series of behavioral tests, microdialysis, and whole-cell recordings on rats exposed to MS to assess the effect of ELS on the relationship between the E/I imbalance of the mPFC and its behavioral deficits in adolescence and adulthood separately. This study revealed a key role of E/I balance in MS-induced disorders, which suggests potential pathogenic mechanisms and therapeutic strategies for ELS.

## Materials and Methods

### Animals

We obtained 8-week-old male and nulliparous female Wistar rats from the Guangdong Laboratory Animals Monitoring Institute. Rats were housed in transparent plastic cages (42 × 26 × 15 cm) under controlled temperatures (22 ± 2°C), humidity (50 ± 5%), and 12 h light-dark cycle. Rats were provided free access to food and water. Following a week of acclimatization, we placed one male rat and one female rat in a cage. Once the female rates were pregnant, they were kept separate from the others. The day of birth was named as postnatal day 0 (P0). Wistar dams and their litters were assigned either to control group or to MS groups. Each experiment was performed according to the procedures outlined by the Regulations for the Administration of Affairs Concerning Experimental Animals (China), and each experiment was reviewed and authorized by the Guangzhou University of Traditional Chinese Medicine Animal Ethics Committee.

### Maternal Separation

“Home separation” maternal separation was performed as reported (Daskalakis et al., 2011; Wang et al., 2015; Banqueri et al., 2017). Simply, “home separation” conducted daily from P1 to P20 for 4 h commencing between 9 am to 1 pm. The dams were daily removed into another cage and the litters remained in the home cage. During the separation, litters were maintained in heating plate at 27°C and water was provided to maintain temperature and humidity. In the control groups, the litters and dams were not disturbed until they were weaned. Each pup was weaned once it reached P21, while four to five males were housed in a single cage until they reached adolescence or adulthood. Females were eliminated.

### Behavior Analysis

For all behavioral tests, rats were habituated to testing room for 30 min prior to experimentation. All behavioral tests were conducted during the animals’ light cycle. In addition to the sucrose preference test (SPT), other tests were recorded by video analysis system (Shanghai Jiliang Software Technology Co., Ltd., Shanghai, China). All tests were conducted double blinded.

#### Open Field Test

The rats were placed in the center of an open-field box (80 cm × 80 cm × 40 cm) and allowed to explore freely for 10 min. Total distance and time spent in the center were recorded by the camera device above the central area of the open field. Total distance and time in the central area were indicators of locomotion activity and anxiety-like behavior. Boxes were cleaned with 75% ethanol before each test.

#### Elevated-Plus-Maze Test

The elevated-plus-maze test (EPMT) was used to evaluate anxiety-like behaviors in rodents. The elevated maze consisted of two open arms (46 cm × 15 cm) and two closed arms (46 cm × 15 cm × 46 cm) was 50 cm above the ground. In the central area of the elevated maze, where the open arm and the closed arm intersect, was equipped with a collection camera. Rat forelimbs simultaneously enter a region referred to as the rats in the region. The rat was placed in the central area with the head facing the same open arm and allowed to move freely for 5 min. The time spent in the open arms were recorded.

#### Novelty Suppressed Feeding Test

After food deprived for 24 h, the rats were placed in the corner of a novel area (80 cm × 80 cm × 40 cm). There was a small (2g) feed in the center, and the latency of rats to pick up the feed and eat were calculated under the camera system for 10 min. The arena was cleaned with 75% ethanol. Testing was followed by a 30 min home cage feeding test to exclude the influence of appetite differences on eating time.

#### Sucrose Preference Test

In the 4-day experiment, each rat was kept in a single cage with two bottles: one contained 1% sucrose water and the other contained water. In the first day, the tested rats were given two bottles of the same 1% sucrose water for 24 h. On the second day, the tested rats were given two bottles of water for 24 h. On the third day, all rats were prohibited from water for 24 h. The fourth day was the test period. Rats were given a bottle of 1% sucrose water and a bottle of water. In order to eliminate the influence of location preference on the results of the experiment, at the 12th hour of the test, the location of sucrose bottle and water bottle was switched. Bottles weighing records before and after the test, and based on the weighing data, calculate the sucrose preference = 100 × [sucrose/(sucrose + water)].

#### Forced Swimming Test

The rat was placed into a cylindrical container (20 cm in diameter, 45 cm in height) which was filled with 30 cm deep water (23 ± 1°C). Put the rat into the transparent plastic cylinder to swim for 6 min, and record the immobility and struggle time of the rat during the last 4 min of the 6 min. Immobility time for the rat defined as a lack of activity except that necessary to keep the head above water.

### Electrophysiological Recording in Acute Slices

#### Slices Preparation

To record the L5 pyramidal neurons from PrL, acute mPFC slices were prepared according to the procedure previously described (Yin et al., 2013). The rats were decapitated to obtain mPFC slices (300 μm). These were processed with a Vibroslice (VT 1200S; Leica) in cold-temperature artificial cerebrospinal fluid (ACSF). The slice-cutting solution contained (in mM): 2.5 KCl, 2.5 MgSO_4_, 1 NaH_2_PO_4_, 26 NaHCO_3_, 1.3 CaCl_2_, 220 sucrose, and 10 D-glucose, and continuously bubbled with 95% O_2/_5% CO_2_. After cutting, the mPFC slices were maintained for recovery for 30 min at 34°C and 1 h at room temperature (25 ± 1°C) in recording ACSF (in mM) 126 NaCl, 3KCl, 1.25 NaH_2_PO_4_, 1MgSO_4_, 2CaCl_2_, 26 NaHCO_3_, and continuously bubbled with 95% O_2_/5% CO_2_.

#### Whole-Cell Patch Clamp Recordings

After recovery for 1 h in ACSF at 32–34°C, the slices were placed in a recording chamber and continuously perfused with ACSF at the rate of 3 ml/min. We conducted whole-cell recordings in the PrL of the L5 pyramidal neurons with an upright microscope, a 40x water-immersion lens (Nikon FN1, Tokyo), and a CCD camera capable of detecting infrared light. The action potential (AP) was recorded in the current-clamp mode and inject a series of step pulses ranging from –200 pA- + 500 pA in a 50 pA increment for 500 ms each, a total of 15 incremental currents were recorded. Input resistance was calculated by linear regression of the slope of the I/V curve after the hyperpolarized current injection (–100 pA) reaches the plateau (Jamann et al., 2021). Patch electrodes (3–5 MΩ) were filled with a solution containing the following (in mM): 105 K-gluconate, 30 KCl, 10 HEPES, 5 EGTA, 4 Mg-ATP, 0.3 Na-GTP, 10 phosphocreatine (pH 7.3, 280 mOsm). For spontaneous excitatory current (sEPSC) and spontaneous inhibitory postsynaptic current (sIPSC) recording, the same cell was respectively clamped at –60 mV and +10 mV and pipettes were filled with an intracellular solution containing (in mM):125 cesium methanesulfonate,10 HEPES, 1 MgCl_2_, 5 CsCl, 0.2 EGTA, 0.3 Na-GTP, 10 phosphocreatine, 4 Mg-ATP and 5 Qx314 (pH 7.3, 280 mOsm). For evoked EPSC (eEPSC) recordings, a stimulating electrode was placed at the border of layer II/III (L2/3) in the PrL, and the recording electrode was positioned sequentially at the L5 in the PrL. Voltage-clamp mode was used for eEPSC recordings (holding potential, –70 mV). The eEPSC was measured in different interpulse intervals of 50, 100, 150, and 200 ms. The stimulus intensity for evoked baseline was 50% maximal response of eEPSC (Swartzwelder et al., 2017). Patch electrodes (3–5 MΩ) were filled with a solution containing the following (in mM):125 cesium methanesulfonate,10 HEPES, 1 MgCl_2_, 5 CsCl, 0.2 EGTA, 0.3 Na-GTP, 10 phosphocreatine, 4 Mg-ATP and 5 Qx314 (pH 7.3, 280 mOsm). In addition to ACSF, the recording fluid must also be added with GABA_A_ receptor antagonist-Bicuculline methiodide (20 μ M). All recordings were performed using an Axon Multiclamp 700B amplifier and Digidata1550B digitizer (Molecular Devices, CA, United States). In all experiments, cells would be excluded if series resistance fluctuated more than 20% of initial values. Signals were sampled at 10 kHz and filtered at 1 kHz.

### Microdialysis

Microdialysis were performed as previously described (Van Schoors et al., 2015). Briefly, a guide cannula (MAB 2/6/9.14.IC, MAB/Microdialysis) was implanted into the right mPFC according to the atlas of Paxinos and Watson [Adolescence: anteroposterior = + 2.60 mm; dorsoventral = 1.80 mm; mediolateral = 0.05 mm; Adult: anteroposterior = + 3.00 mm; dorsoventral = 2.00 mm; mediolateral = 0.05 mm; relative to bregma]. A microdialysis probe (MAB 6.14.2, membrane length: 2 mm, molecular weight cut-off: 15,000 Da, outer diameter: 0.6 mm) was inserted through the guide cannula and then connected the probe to the syringe pump (BASi MD-1001). The probes were perfused with modified Ringer’s solution (composition in mM: NaCl 147, KCl 4, CaCl_2_-2H_2_O 2.2, pH = 7) at a constant flow rate of 2 μl min^–1^. After 60 min of equilibrium between tissue and perfusion liquid, three samples (40 μl each) were automatically collected from each rat using the microfraction collector (BASi small fc) every 20 min over 60 min under 4°C. The samples were merged and subsequently stored at –80°C until analyzed. The locations of the microdialysis probes were verified using 40 mm-thick coronal cryostat sections at the end of the study.

### Glutamate and γ-Aminobutyric Acid Analysis

#### Sample Preparation

We processed the internal standard (IS) isoprenaline, as well as stock solutions of neurotransmitters, by dissolving a pre-determined amount in a solution (formic acid: water: methanol = 2: 800: 200, v/v) until we obtained the following concentrations: 1 mg/mL of isoprenaline, 4 mg/mL for γ-aminobutyric acid (GABA), and 2 mg/mL for glutamate. The stock solutions were kept at –80°C for subsequent use. We established calibration protocols by mixing each stock solution (which was diluted) with acetonitrile (obtain IS 200 ng/mL) until we reached eight calibration standards with concentration intervals of 100–20000 ng/mL for GABA and 50–10000 ng/mL for glutamate. We prepared the control samples the same way, which produced three corresponding QCs: 300, 2500, and 15000 ng/mL for GABA; 150, 1250, and 7500 ng/mL for glutamate. Dialyzate, mix solution and acetonitrile (obtain IS 200 ng/mL) (19:1:40, v/v) were added to a 2.0 mL Eppendorf^®^ tube, which was closed and agitated for 30 s in a vortex mixer. Then, the mixture was centrifuged at 13,000 rpm and 4.0°C for 20 min for protein precipitation. The upper organic phase, which used for analysis by high performance liquid chromatography-mass spectrometry (HPLC-MS), was collected.

#### High Performance Liquid Chromatography-Mass Spectrometry Analysis

In the dialyzate, GABA and glutamate were measured by HPLC with mass spectrometry. The HPLC processes used a 1200 Series separation module (Agilent Technologies, Santa Clara, United States) and an Agilent Mass Hunter Workstation Data Acquisition software to program the chromatographic conditions and the samples. The separations were performed at a temperature of 25°C in ACE3 C18-PFP (150 × 4.6 mm, 3 μm particle size), which was protected with a 0.2-μm on-line filter. The mobile phase consisted of a combination of acetonitrile (solution A) and 0.2% formic acid in MilliQ water (solution B). The flow rate was 0.6 mL/min. The chromatogram was run under gradient conditions as follows: 0–2 min at 5% (A) and 95% (B); 2–5 min, gradually decreasing eluent B to 10%; 5–9.5 min, 10% (B); 9.5–17 min, 95% (B). The run time was 9.5 min followed by 6.5 min of recalibration, resulting in 17 min of total run time. Ten μL was injected into the chromatographic system. For the mass spectrometry detection system, an Agilent Technologies 6410 triple quadrupole (Santa Clara, United States) set to multiple reaction monitoring (MRM) mode was used, along with an electrospray ionization (ESI) source set to positive ion mode. The ESI parameters were as follows: Nebulizer pressure 30 PSI, flow 12 L/min, capillary voltage 4000 V, and desolvation gas temperature (Nitrogen) 325°C. The peak area was measured relative to isoprenaline (Supplementary Table 1). We used the Mass Hunter Workstation Quantitative Analysis software (Agilent Technologies, Santa Clara, Unites States) to assess the integration peak area of the MRM transitions for each analyte.

### Statistical Analysis

All of results are expressed as the means ± SEM. The statistical analyses were performed using SPSS (version 26.0). Data from each group was assessed for normality by using the Shapiro–Wilk Test. A two-tailed Mann–Whitney *U*-test was used to evaluate statistical significance in non-parametric tests. If data was found to be normal, then differences between two groups were analyzed using independent sampled *t*-tests unless otherwise noted. Electrophysiological studies used two-way repeated measures ANOVA analyses, including PPR and AP frequency. Welch’s correction was performed only if the analysis of variance was found to have a significant difference. *P* < 0.05 was considered statistically significant for all tests.

## Results

### Maternal Separation Induces Different Behaviors in Adolescent and Adult Rats

**FIGURE 1:**
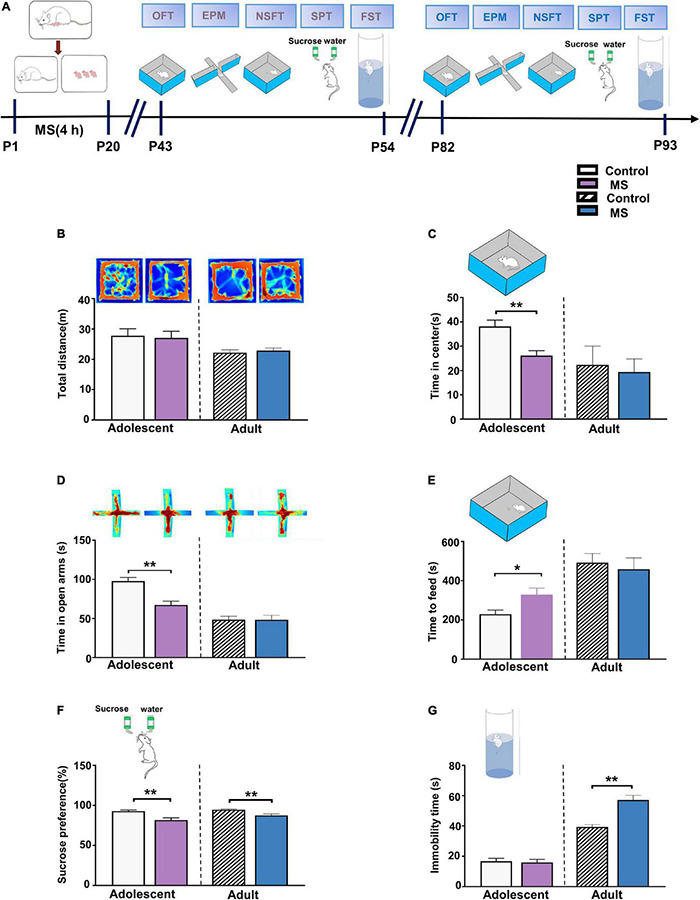
Maternal separation induces different behaviors in adolescent and adult rats. **(A)** The experimental timeline of MS, behavioral tests. **(B)** Upper: representative traces of the movement of adolescent and adult rats in the open field experiment. Lower: the total distance in the open field experiment of adolescent and adult rats, respectively. **(C)** MS decreased the time spent in the center area compared to control rats in adolescent, while there was no difference between MS and control in adulthood. **(D)** Upper: representative traces of the movement of adolescent and adult rats in the Elevated-Plus-Maze Test. Lower: compared to Control groups, MS induces a decrease of the time in open arms for adolescent rats, but no change in rats with MS compared to control rats in adult. **(E)** MS increased the latency of time to feed compared to control rats in adolescent, while there was no difference between MS and control in adulthood. **(F)** Reduced sucrose preference rate by rats with MS in SPT test compared to control group rats whether in adolescence or adulthood. **(G)** Immobility time in rats with MS compared to control rats was no change of adolescent rats in the forced swimming test, but induce an aggravation in immobility time in rats with MS compared to control rats in adult. (Adolescent: *n* = 15 rats each group; Adult: *n* = 12 rats each group) *p < 0.05, **p < 0.01. Data are the means ± SEM.

To test the effect of MS on anxiety-like behaviors at different developmental stages, we conducted series behavioral experiments including open field test (OFT), EPMT, and novelty suppressed feeding test (NSFT) (Calhoon and Tye, 2015; de Carvalho et al., 2021; Qin et al., 2021). SPT and Forced Swimming Test (FST) were performed to observe depressive-like behavior, which related to two core symptom domains in depression (Berton et al., 2012; Ramaker and Dulawa, 2017; Figure 1A). In adolescence, we found that the MS rats showed no difference in total distance (*p* = 0.756, Mann–Whitney *U*-test, Figure 1B) but showed decreased time in the central field (*t* = 3.579, *p* = 0.0013, unpaired *t*-test, Figure 1C) compared with the control rats. Furthermore, the MS rats spent less time in the open arms in the EPMT (*t* = 4.419, *p* = 0.0001, unpaired *t*-test, Figure 1D) and needed more time to feed in NSFT (*t* = 2.556, *p* = 0.0163, unpaired *t*-test, Figure 1E) than the control rats. In addition, the MS rats exhibited a significantly reduced sucrose consumption rate in SPT compared with the control rats (*p* = 0.003, Mann–Whitney U-test, Figure 1F). However, MS did not affect the immobility time (*t* = 0.273, *p* = 0.787, unpaired *t*-test, Figure 1G). Collectively, these results indicate that MS induces both anxiety- and depressive-like behaviors in adolescent rats.

In adulthood, however, in the OFT, there were no differences in the total distance (*t* = 0.5268, *p* = 0.6036, unpaired *t*-test, Figure 1B) or in the central time (*p* = 0.686, Mann–Whitney *U*-test, Figure 1C) between the control and MS rats. Furthermore, there was no difference between the control and MS rats during the time in the open arms in EPMT and while feeding in NSFT (EPMT: *t* = 0.026, *p* = 0.980, unpaired *t*-test; NSFT: *p* = 0.507, Mann–Whitney *U*-test, Figures 1D,E). To further determine whether MS could cause depressive-like behavior in adults, we measured SPT and FST. As shown in Figure 1F, the MS rats exhibited a decreased sucrose consumption rate in SPT compared with the control rats, indicating an anhedonia-like behavior in the MS rats (SPT: *p* = 0.006, Mann–Whitney *U*-test). Similarly, in another behavioral test of depression in FST, the MS rats showed a considerably increased immobility time compared with the control rats (*p* = 0.001, Mann–Whitney *U*-test, Figure 1G). Taken together, these results show that MS induces depressive-like behaviors but not anxiety-like behaviors in adulthood in rats.

### Maternal Separation Induces an Increase in Neuronal Excitability in L5 Pyramidal Cells in Adolescence

**FIGURE 2:**
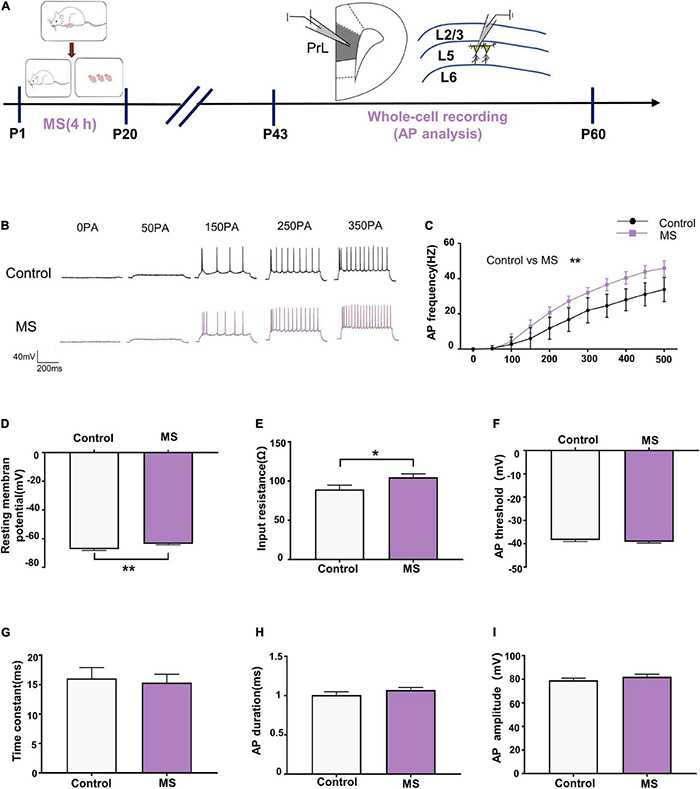
Maternal separation induces an increase in neuronal excitability in L5 pyramidal cells in adolescence. **(A)** The experimental timeline of MS, whole-cell recording (AP analysis). **(B)** Representative trace of AP from the L5 pyramidal cell in PrL with current clamp under the condition of injecting incremental step currents of 0, +50, +150, +250, and +350 pA in control and MS at adolescent. Scale bars: 40 mV, 200 ms. **(C)** The frequency of action potential firing of pyramidal neurons in control and MS at adolescent. **(D)** The resting membrane potential of action potential of pyramidal neurons in control and MS at adolescent. **(E)** The input resistance of action potential of pyramidal neurons in control and MS at adolescent. **(F)** The threshold of action potential of pyramidal neurons in control and MS at adolescent. **(G)** The membrane time constant of action potential of pyramidal neurons in control and MS at adolescent. **(H)** The duration of the action potential measured at half-amplitude of pyramidal neurons in control and MS at adolescent. **(I)** The amplitude of action potential of pyramidal neurons in control and MS at adolescent. *p < 0.05, **p < 0.01. Data are the means ± SEM (*n* = 14 cells from 3 to 4 rats each group).

As principal output neurons to subcortical mPFC regions, L5 pyramidal neurons in the PrL reportedly contribute to the development of psychiatric disorders (Shi et al., 2019). To further determine the mechanism underlying the different behavioral phenotypes in different life periods, we performed whole-cell recordings to analyze the firing of L5 pyramidal neurons in the PrL region from P43 to P60 (Figure 2A). As shown in Figure 2C, compared with the control rats, the AP frequency was significantly increased in the mPFC of the MS rats in adolescence [repeated-measures two-way ANOVA: time, *F*_(10_,_260)_ = 576.2, *p* < 0.0001; group, *F*_(1_,_26)_ = 36.76, *p* < 0.0001; interaction, *F*_(10_,_260)_ = 16.78, *p* < 0.0001]. Meanwhile, both the AP resting membrane potential and input resistance were significantly increased in the mPFC of the MS rats compared with those in the control rats (resting membrane potential: *t* = 3.236, *p* = 0.0033, unpaired *t*-test; input resistance: *t* = 2.221, *p* = 0.0353, unpaired *t*-test, Figures 2D,E). However, there was no difference between the control and MS rats in terms of AP threshold, time constant, half-amplitude duration, and amplitude (threshold: *t* = 0.9246, *p* = 0.3637, unpaired *t*-test; time constant: *p* = 0.963, Mann–Whitney *U*-test; half-amplitude duration: *p* = 0.111, Mann–Whitney *U*-test; amplitude: *p* = 0.129, Mann–Whitney *U*-test; Figures 2F–I). These results indicate that MS-induced anxiety- and depressive-like behaviors in adolescence may be associated with the excessive excitability of L5 pyramidal neurons in the PrL.

### Maternal Separation Induces a Decrease in Neuronal Excitability in L5 Pyramidal Cells in Adulthood

**FIGURE 3:**
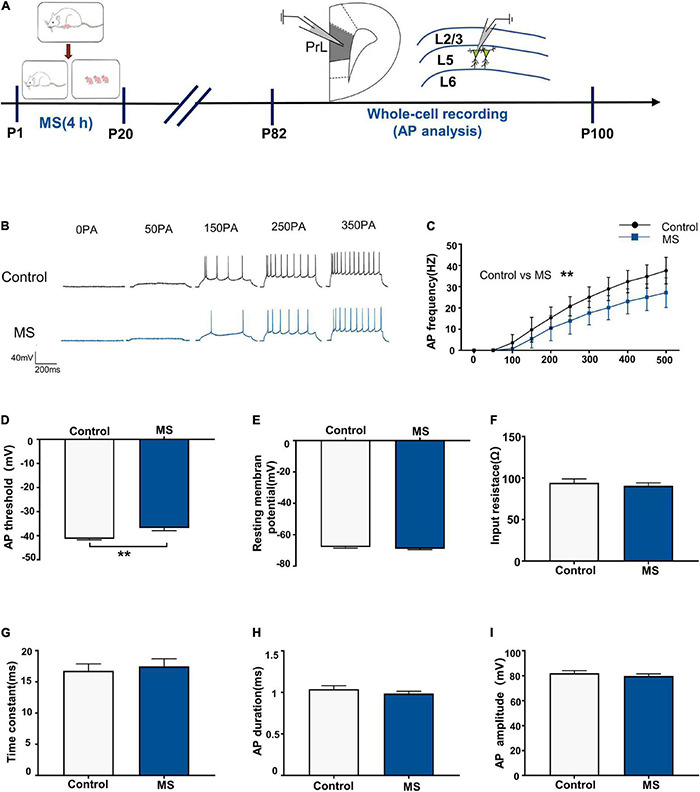
Maternal separation induces a decrease in neuronal excitability in L5 pyramidal cells in adulthood. **(A)** The experimental timeline of MS, whole-cell recording (AP analysis). **(B)** Representative trace of AP from the L5 pyramidal cell in PrL with current clamp under the condition of injecting incremental step currents of 0, +50, +150, +250, and +350 pA in control and MS at adult. Scale bars: 40 mV, 200 ms. **(C)** The frequency of AP firing of pyramidal neurons in control and MS at adult. **(D)** The threshold of action potential of pyramidal neurons in control and MS at adult. **(E)** The resting membrane potential of action potential of pyramidal neurons in control and MS at adult. **(F)** The input resistance of action potential of pyramidal neurons in control and MS at adult. **(G)** The membrane time constant of action potential of pyramidal neurons in control and MS at adult. **(H)** The duration of the action potential measured at half-amplitude of pyramidal neurons in control and MS at adult. **(I)** The amplitude of action potential of pyramidal neurons in control and MS at adult. *p < 0.05, **p < 0.01. Data are the means ± SEM (*n* = 15 cells from 3 to 4 rats each group).

Next, we performed whole-cell recordings of L5 pyramidal neurons in the PrL region at P82–P100 to determine the mechanism underlying the depressive-like behavioral deficits in adult rats induced by MS (Figure 3A). There was a significantly lower AP firing frequency in L5 neurons compared with that in the control rats [repeated-measures two-way ANOVA: time, *F*_(10_,_280)_ = 385.6, *p* < 0.0001; group, *F*_(1_,_28)_ = 18.84, *p* = 0.0002; interaction, *F*_(10_,_280)_ = 9.311, *p* < 0.0001, Figures 3B,C]. Intriguingly, the AP threshold in L5 neurons in the MS rats was higher than that in the control rats (*p* = 0.006, Mann–Whitney *U*-test, Figure 3D). However, there was no difference in resting membrane potential, input resistance, time constant, half-amplitude duration, and amplitude between the control and MS rats (resting membrane potential: *t* = 1.077, *p* = 0.2908, unpaired *t*-test; input resistance: *t* = 0.6092, *p* = 0.5473, unpaired *t*-test; time constant: *p* = 0.917, Mann–Whitney *U*-test; half-amplitude duration: *p* = 0.521, Mann–Whitney *U*-test; amplitude: *t* = 0.8366, *p* = 0.4099, unpaired *t*-test; Figures 3E–I). The above results indicate that MS-related depressive-like behaviors in adult rats may be due to the decreased excitability of L5 pyramidal neurons in the mPFC.

### Increased Excitation/Inhibition Balance of L5 Pyramidal Cells in Adolescent Maternal Separation Rats

**FIGURE 4:**
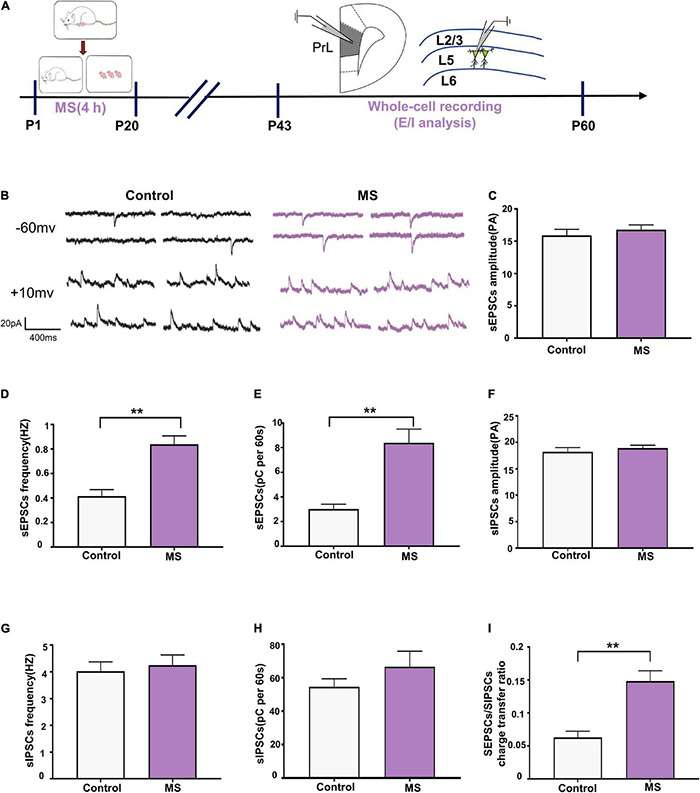
Increased E/I balance of L5 pyramidal cells in adolescent MS rats. **(A)** The experimental timeline of MS, whole-cell recording (E/I analysis). **(B)** Representative sEPSC (upper) and sIPSC (lower) current traces at a holding potentiation of –60 and + 10 mV, respectively. Scale bars: 20 pA, 400 ms. **(C–E)** Changes in the amplitude, frequency, and quantification of sEPSC current recorded by isolated patch clamp in adolescent rats. **(F–H)** Changes in the amplitude, frequency, and quantification of sIPSC current recorded by isolated patch clamp in adolescent rats. **(I)** sEPSC/sIPSC charge transfer ratios. *p < 0.05, **p < 0.01. Data are the means ± SEM (*n* = 18 cells from 3 to 4 rats each group).

To determine whether the altered balance between excitatory and inhibitory synaptic transmission affects the excitability of pyramidal cells, we assessed the sEPSC/sIPSC charge transfer (E/I) ratio of L5 pyramidal cells in the PrL in mPFC slices from adolescent rats (Figure 4A). Both sEPSCs and sIPSCs were recorded from the same L5 pyramidal cells in the PrL region at holding potentials of –60 mV and +10 mV for P43–P60 (Figure 4B). sEPSC recordings showed that the MS rats exhibited an increased frequency and quantity of charge compared with the control rats (sEPSC frequency: *t* = 4.844, *p* < 0.0001, unpaired *t*-test; sEPSC quantification: *p* < 0.0001, Mann–Whitney *U*-test; Figures 4D,E) but not amplitude (sEPSC amplitude: *p* = 0.569, Mann–Whitney *U*-test; Figure 4C). Unlike with sEPSCs, MS does not affect the frequency, amplitude, and quantity of charge of sIPSCs in L5 pyramidal neurons in adolescence (sIPSC amplitude: *p* = 0.107, Mann–Whitney *U*-test; sIPSC frequency: *t* = 0.4432, *p* = 0.6604, unpaired *t*-test; sIPSC quantification: *p* = 0.681, Mann–Whitney *U*-test; Figures 4F–H). To further determine the balance of excitatory and inhibitory synaptic currents onto pyramidal cells, the sEPSC/sIPSC charge transfer ratio was analyzed. As shown in Figure 4I, the MS rats had a significantly increased sEPSC/sIPSC charge transfer ratio compared with the control rats (*p* < 0.0001, Mann–Whitney *U*-test). The above results suggest that MS increases the E/I balance of synaptic transmission onto L5 pyramidal neurons in the PrL of adolescent rats.

### Decreased Excitation/Inhibition Balance of L5 Pyramidal Cells in Adult Maternal Separation Rats

**FIGURE 5:**
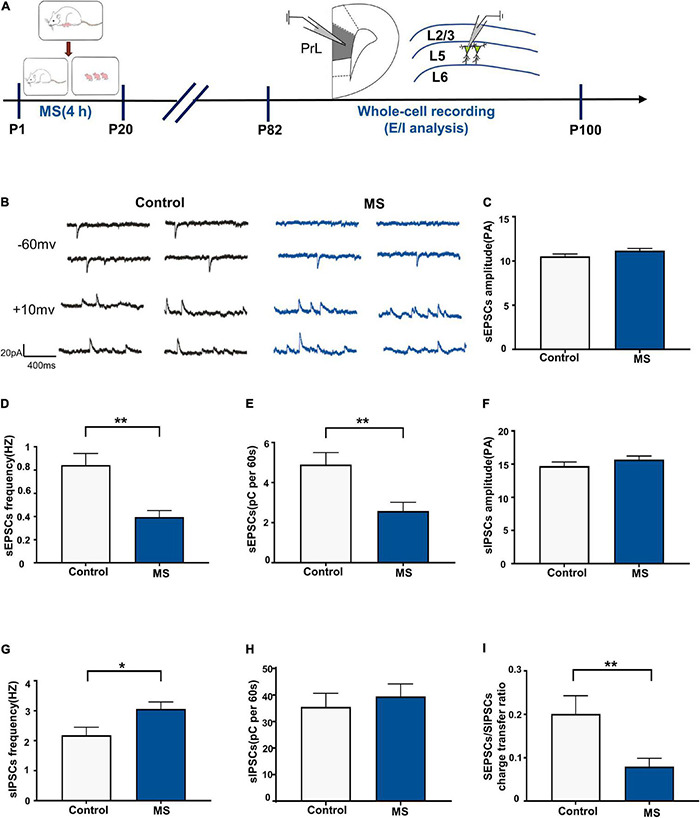
Decreased E/I balance of L5 pyramidal cells in adult MS rats. **(A)** The experimental timeline of MS, whole-cell recording (E/I analysis). **(B)** Representative sEPSC (upper) and sIPSC (lower) current traces at a holding potentiation of –60 and +10 mV, respectively. Scale bars: 20 pA, 400 ms. **(C–E)** Changes in the amplitude, frequency, and quantification of sEPSC current recorded by isolated patch clamp in adult rats. **(F–H)** Changes in the amplitude, frequency, and quantification of sIPSC current recorded by isolated patch clamp in adult rats. **(I)** sEPSC/sIPSC charge transfer ratios. *p < 0.05, **P < 0.01. Data are the means ± SEM (*n* = 14–15 cells from 3 to 4 rats each group).

We performed whole-cell recordings of L5 pyramidal neurons in the PrL region to analyze sEPSCs and sIPSCs for P82–P100 (Figure 5A) and found that the frequency and quantity of charge, but not amplitude, of sEPSCs were significantly decreased in the MS rats compared with those in the control rats (sEPSC amplitude: *t* = 1.848, *p* = 0.0755, unpaired *t*-test; sEPSC frequency: *p* = 0.001, Mann–Whitney *U*-test; sEPSC quantification: *t* = 3.078, *p* = 0.0047, unpaired *t*-test; Figures 5C–E). Notably, MS increases sIPSC frequency (*t* = 2.455, *p* = 0.0208, unpaired *t*-test, Figure 5G) but not amplitude (*t* = 1.167, *p* = 0.2535, unpaired *t*-test, Figure 5F) or quantity of charge (*t* = 0.5695, *p* = 0.5737, unpaired *t*-test, Figure 5H) in the MS rats compared with those in the control rats. Consistent with the changes in sEPSC and sIPSC frequency, MS resulted in a significant decrease of the sEPSC/sIPSC charge transfer ratio (*p* = 0.009, Mann–Whitney *U*-test, Figure 5I). These results suggest that MS decreases the E/I balance of L5 pyramidal neurons in the PrL of adult rats.

### Maternal Separation Induces the Expression of Different Glutamate Levels in Rat Prelimbic in Adolescence and Adulthood

**FIGURE 6:**
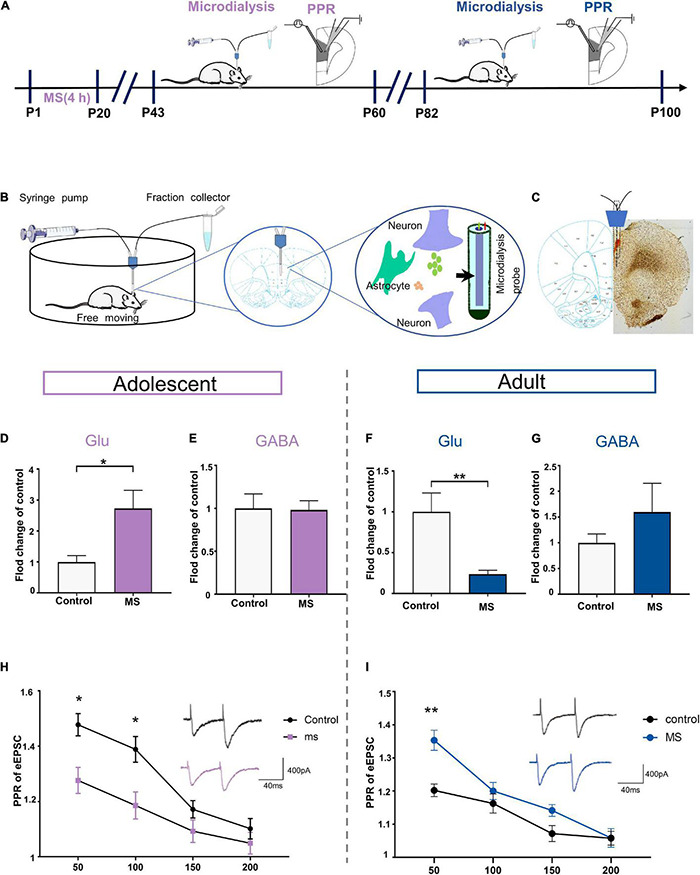
Maternal separation induces the expression of different glutamate levels in rat PrL in adolescence and adulthood. **(A)** The experimental timeline of MS, microdialysis and whole-cell recording (PPR analysis). **(B)** Schematic diagram of microdialysis experiment flow. **(C)** A coronal view showing the microdialysis probe at prelimbic cortex. **(D,E)** Represents the changes of glutamate and GABA level in mPFC recorded by microdialysis *in vivo* in adolescent rats (*n* = 6–7). **(F,G)** Represents the changes of glutamate and GABA level in mPFC recorded by microdialysis *in vivo* in adult rats (*n* = 5–6). **(H)** PPR evoked by paired stimuli (interstimulus interval: 50, 100, 150, and 200 ms) in mPFC L2/3-L5 synapses for control and MS in adolescent (*n* = 15 cells, 3–4 rats each group). Right: Representative traces PPR with interstimulus interval of 50 ms for control and MS in adolescent. Scale bars: 400 pA, 40 ms. **(I)** PPR evoked by paired stimuli (interstimulus interval: 50, 100, 150, and 200 ms) in mPFC L2/3- L5 synapses for control and MS in adult (control: *n* = 3–4 rats, cell = 15; MS: *n* = 3–4 rats, cell = 20). Right: Representative traces PPR with interstimulus interval of 50 ms for control and MS in adolescent. Scale bars: 400 pA, 40 ms. *p < 0.05, **p < 0.01. Data are the means + SEM.

To determine whether the extracellular levels of glutamate and GABA in the mPFC affect the E/I balance of L5 pyramidal cells in the PrL, we employed *in vivo* microdialysis techniques coupled to HPLC with mass spectrometry in free moving rats in adolescence and adulthood, respectively (Figures 6A,B). Microscopic examination was employed to determine the correct positioning of the embedded sleeve in the PrL (Figure 6C). The MS rats exhibited higher extracellular glutamate levels in the interstitial fluid of the mPFC than the control rats in adolescence, whereas the GABA levels did not differ between the rat groups (Glu: *t* = 2.588, *p* = 0.0252, unpaired *t*-test; GABA: *t* = 0.095, *p* = 0.926, unpaired *t*-test; Figures 6D,E). However, the glutamate level was significantly decreased in the MS rats compared with that in the control rats in adulthood (*p* = 0.0063, Mann–Whitney *U*-test, Figure 6F). Although not statistically significant, the MS rats in adulthood exhibited higher extracellular GABA levels than the control rats (*t* = –1.013, *p* = 0.351, unpaired *t*-test, Figure 6G).

Considering that the altered extracellular glutamate levels can be affected by increasing the release of transmitters, we determined the probability of presynaptic glutamate release through the evoked EPSC (eEPSC) paired-pulse ratio (PPR) of pyramidal neurons in the PrL. Compared with that in the control rats, the PPR was significantly lower at interstimulus intervals of 50 and 100 ms in the MS rats in adolescence [repeated-measures two-way ANOVA: time, *F*_(3_,_84)_ = 33.207, *p* < 0.0001; group, *F*_(1_,_28)_ = 10.295, *p* = 0.0033; interaction, *F*_(3_,_84)_ = 2.736, *p* = 0.0486, Figure 6H]. In contrast, we found higher PPR in the adult MS rats than in the control rats at an interstimulus interval of 50 ms [repeated-measures two-way ANOVA: time, *F*_(3_,_96)_ = 32.253, *p* < 0.0001; group, *F*_(1_,_32)_ = 13.873, *p* = 0.001; interaction, *F*_(3_,_96)_ = 3.463, *p* = 0.019, Figure 6I]. Together, these observations indicate that abnormal presynaptic glutamate release may contribute to the different glutamate levels observed in adolescence and adulthood in rats exposed to MS.

## Discussion

Maternal separation is one of the most frequently used animal models in the study of ELS because it stably simulates the impact of the lack of maternal care during early development on the offspring’s physical and psychological development (O’Mahony et al., 2011; Tractenberg et al., 2016). In our MS model, rats exhibited anxiety-like behaviors in adolescence (Figures 1B–E), which is consistent with other studies that used the same animal model (Banqueri et al., 2017; Auth et al., 2018). Studies have shown that MS results in an elevated stress response and depressive-like behavior in adolescent rats, which is consistent with our study (Yang et al., 2016; Hu et al., 2017; Kim H. B. et al., 2020; Abdelwahab et al., 2021). Additionally, our results showed that MS rats exhibited depressive-like behavior in adulthood (Figures 1F,G), which confirmed the findings of previous studies showing that MS leads to depression in adulthood (Arborelius et al., 2004; Vetulani, 2013; Masrour et al., 2018; Ruiz et al., 2018). Considering that the MS model is a neurodevelopmental model, we speculated that different behaviors in adolescence and adulthood in the same biological individuals may be attributable to variations in neurodevelopmental processes sensitive to ELS. Previous studies using rodent models also found that ELS could result in more varying phenotypes in childhood than in adolescence (Raineki et al., 2012; Rincon-Cortes and Sullivan, 2014), which provide evidence to support our speculation. Because the formation and connection of neurodevelopmental processes are mainly established from the embryonic period through adolescence, external and internal factors can affect the function of these processes and thus the behaviors they induce (Suri et al., 2015). However, at different stages of development, individual neurodevelopmental processes can result in different susceptibility to psychological disorders such as anxiety and depression, and this may lead to different physiological response trajectories or behavioral characteristics (Jawahar et al., 2015). Moreover, previous evidence suggests that the effects of early life adversity differ between sexes (Goodwill et al., 2019; Honeycutt et al., 2020). ELS increases the susceptibility to stress in female mice in adulthood (Pena et al., 2019). However, our current study only focused on behaviors in males, but we will conduct future studies on the impact of MS in different genders.

Early life stress has been shown to impair mPFC function later in life (Youssef et al., 2019). Changes in glutamatergic and GABAergic transmission have been observed not only in adults with depression (Rajkowska et al., 2007) but also in animals exposed to chronic stress (Page and Coutellier, 2018; Mackowiak et al., 2019). In our study, electrophysiological recordings revealed that ELS increased the E/I balance in mPFC pyramidal cells in adolescence (Figure 4). Another study also demonstrated that MS increased the expression of glutamate receptors 1 and 2, Ca^2+^/calmodulin-dependent protein kinase II, and postsynaptic density protein 95 in the mPFC in adolescent rats, which exhibited increased anxiety-like behavior (Chocyk et al., 2013). Furthermore, our study revealed that ELS decreases the E/I balance in mPFC pyramidal cells in adulthood (Figure 5). Collectively, these results provide further evidence that the E/I balance may play a key role in early stress-induced anxiety and depression.

Glutamate is the most crucial excitatory neurotransmitter in the brain and it plays a key role in stress-related diseases such as depression and anxiety (Lim et al., 2012; Pitsikas, 2014). Importantly, the current study showed that MS induced anxiety-like behavior with increased glutamate levels in adolescence (Figures 2, 6) but resulted in depressive-like behavior with decreased glutamate levels in adulthood (Figures 3, 6). Similarly, the weakened synaptic transmission of mPFC excitatory glutamate is believed to be related to depression (Thompson et al., 2015). Furthermore, *in vivo* microdialysis combined with HPLC-MS analysis showed that the glutamate levels in the brain and cerebrospinal fluid of patients with depression were significantly reduced (Frye et al., 2007). Consistent with our conclusion, glutamatergic agents have common anti-anxiety and anti-depressive effects. For example, ketamine causes a burst of mPFC glutamate, leading to BDNF release and TrkB-Akt stimulation, thereby activating mTORC1 signaling, which leads to increased protein synthesis required for synapse maturation and formation and the generation of a rapid anti-depressive response (Berman et al., 2000; Zarate et al., 2006; Duman et al., 2016). However, our findings only focused on changes in the E/I balance of the adolescent and adult periods. Since only a few studies have addressed the alteration of E/I balance at different developmental stages, the process by which glutamate and GABA participate in the regulation of E/I balance requires further study.

Nevertheless, the pathways by which MS impact behavior and the mechanisms of various neural activities during different developmental stages remains elusive. The alteration of multiple genes could be important factors for neural activity induced by MS. For example, alterations of myelin-related genes and immediate early genes were found before adulthood (Teissier et al., 2020). Our RNA-seq data from a previous study showed that genes relevant to syntaxin and SNARE binding (*Syt6*, *Cplx3, Cav2*) were significantly affected by MS stress in adults (Zheng et al., 2019). Additionally, neuronal excitability may contribute to the different behavior caused by MS. MS caused the reduction of excitability in glutamatergic neurons (Qin et al., 2021) and increased neuronal excitability in mPFC can prevent the emergence of depressive-like behavior induced by MS (Teissier et al., 2020). Moreover, neuronal spine structure may also play a specific role. The density of dendritic spines of layer II/III/V pyramidal neurons show losses in mPFC induced by MS (Glaser and Van der Loos, 1981; Chocyk et al., 2013; Franco et al., 2020). Finally, parvalbumin (PV+) interneurons have also been reported to participate in the modulation of E/I balance (Ferguson and Gao, 2018), and MS was shown to reduce PV expression in the PFC at 40 days postnatal (Wieck et al., 2013; Grassi-Oliveira et al., 2016), but not at P100 (Leussis et al., 2012).

In summary, this study provides evidence that different behavioral deficits in adolescents and adulthood caused by MS may be due to an E/I imbalance. MS selectively altered the frequency of sEPSC and sIPSC but had no effect on the amplitude, indicating that the effect of MS on the E/I imbalance was presynaptic. We also found that the abnormal extracellular glutamatergic level in adolescence and adulthood may be a result of presynaptic glutamate release deficits. However, the process by which MS results in abnormal glutamatergic and GABAergic transmission in mPFC warrants further study.

## Data Availability Statement

The original contributions presented in the study are included in the article/Supplementary Material, further inquiries can be directed to the corresponding author/s.

## Ethics Statement

The animal study was reviewed and approved by Animal Experiment Ethics Committees of Guangzhou University of Chinese Medicine.

## Author Contributions

YJC designed the experiments. YWC, SZ, and WL conducted the behavioral test. YWC and CZ conducted the electrophysiology experiments. YZ conducted the microdialysis experiments. YWC, JY, and YZ conducted the data analysis. YJC and YWC wrote the manuscript. LY helped revise the manuscript. All the authors read and approved the final manuscript.

## Conflict of Interest

The authors declare that the research was conducted in the absence of any commercial or financial relationships that could be construed as a potential conflict of interest.

## Publisher’s Note

All claims expressed in this article are solely those of the authors and do not necessarily represent those of their affiliated organizations, or those of the publisher, the editors and the reviewers. Any product that may be evaluated in this article, or claim that may be made by its manufacturer, is not guaranteed or endorsed by the publisher.
