# Supplementary material for: Early Life Stress Induces Different Behaviors in Adolescence and Adulthood May Related With Abnormal Medial Prefrontal Cortex Excitation/Inhibition Balance
Source: Front Neurosci. 2022 Jan 4;15:720286. doi: 10.3389/fnins.2021.720286 (PMC8765554; doi:10.3389/fnins.2021.720286)
Supplement: Supplementary file 1 [file Table_1.pdf]

Supplementary Table 1

Identification of the chemical compounds in MRM mode

Compound	m/z	Fragmentor	Collision Energy
GABA	104.0/45.1	65	22
Glutamate	148.0/84.0	75	13
areoprenaline	463.1/299.8	128	24
